# The relationship between antidepressants and breast cancer: evidence from Mendelian randomization

**DOI:** 10.1007/s10552-023-01766-z

**Published:** 2023-08-04

**Authors:** Dong Niu, Chenchen Li, Xiaoyu Yan, Haoran Qu, Yuling Zheng

**Affiliations:** 1 Henan University of Chinese Medicine, Zhengzhou, China; 2 The First Affiliated Hospital of Henan University of Chinese Medicine, Zhengzhou, China

**Keywords:** Antidepressants, Breast cancer, Mendelian randomization, Cancer risk

## Abstract

**Background:**

The use of antidepressants has increased over the years, but the relationship between antidepressant use and the risk of breast cancer is not uniform because of confounding factors. We aimed to assess the effect of antidepressants on breast cancer risk using a two-sample Mendelian randomization (MR) approach.stet

**Methods:**

Secondary data analysis was performed on pooled data from genome-wide association studies based on single-nucleotide polymorphisms that were highly correlated with antidepressants, SSRI drugs, and serotonin and prolactin levels were selected as instrumental variables to evaluate the association between antidepressants and SSRI drugs and prolactin levels with breast cancer and ER+/ER- breast cancer. We then performed a test of the hypothesis that SSRI drugs elevate prolactin concentrations. We performed two-sample Mendelian randomization analyses using inverse variance weighting, MR-Egger regression, and weighted median methods, respectively.

**Results:**

There was no significant risk association between antidepressant and SSRI use and the development of breast cancer, ER-positive or ER-negative breast cancer (*P* > 0.05), and serotonin concentration was not associated with breast cancer risk (*P* > 0.05). There was a positive causal relationship between prolactin levels and breast cancer (IVW, *P* = 0.02, OR = 1.058) and ER-positive breast cancer (Weighted median, *P* = 0.043, OR = 1.141; IVW, *P* = 0.009, OR = 1.125). Results in SSRI medication and prolactin levels showed no association between SSRI analogs and prolactin levels (*P* > 0.05).

**Conclusion:**

Large MR analysis showed that antidepressants as well as SSRI drugs were not associated with breast cancer risk and the SSRI-prolactin-breast cancer hypothesis did not hold in our analysis.

**Supplementary Information:**

The online version contains supplementary material available at 10.1007/s10552-023-01766-z.

## Introduction

In 2021, the International Agency for Research on Cancer released a report on the incidence and mortality of 36 types of cancer in 185 countries and territories worldwide. The report shows that breast cancer has become the most prevalent malignancy in women worldwide and the leading cause of cancer deaths in women, and that the incidence and mortality of breast cancer in women have been on the rise since the twenty-first century [1]. In the United States, for example, by 2022, approximately 287,850 new cases of invasive breast cancer and 51,400 cases of ductal carcinoma in situ will be diagnosed in American women, and 43,250 women will die from breast cancer [2]. The burden of breast cancer has increased further and in order to reduce the burden of breast cancer, many researchers are actively searching for risk factors for breast cancer in order to reduce the incidence of breast cancer.

The use of antidepressants has increased in most countries and regions in recent decades [3, 4]. In the United States, for example, the use of antidepressants has increased from 10.6 to 13.8% in the last decade, with almost one in seven people taking antidepressants, and the rate of antidepressant use is even higher among women, with almost one in five women using antidepressants and even one in four women over the age of 60 taking antidepressants. The use of antidepressants in Australia and European countries is similar to that in the US [3, 5, 6]. The use of antidepressants can cause a variety of diseases. However, there is no single answer to the question of whether the use of antidepressants increases the risk of breast cancer. Many researchers believe that there is a biological basis for the increased risk of breast cancer from antidepressants. For example, selective serotonin reuptake inhibitors (SSRIs) are commonly prescribed as first-line antidepressants, which exert antidepressant effects by increasing synaptic 5-hydroxytryptamine (5-HT) concentrations [7]. 5-HT acts on prolactin-releasing factor, which increases the level of prolactin-releasing factor and thus increases the concentration of prolactin [8]. All SSRIs increase the basal level of prolactin in the body to a greater or lesser extent, and prolactin concentration is closely related to the proliferation and differentiation of breast cancer cells [9, 10]. Although there are reasons to support that antidepressants may increase the risk of breast cancer, the results of many clinical trials have yielded different results. This may have been confounded by a number of confounding factors, for example, depression and obesity are often combined, and obesity is a high risk factor for breast cancer [11–13]. It may be that people taking antidepressants have high risk factors such as alcohol abuse and high BMI levels that can cause breast cancer [14], which are difficult to avoid.

In epidemiological studies, the presence of confounding factors has greatly confounded causal inferences about exposures and outcomes. Mendelian Randomization (MR) reduces the effects of confounding and is based on the principle that genetic variants are randomly assigned at the time of conception and that one trait is usually uncorrelated with the others. This process is similar to randomly assigning participants to treatment and control groups in a randomized controlled trial [15]. The MR design also minimizes reverse causality, as alleles are fixed at birth and cannot change with the onset or progression of disease. Using genetic variation as an instrumental variable to infer causal associations between exposure and outcome reduces the confounding factors [16]. We therefore intend to use Mendelian randomization to explore whether there is an association between the use of antidepressants and an increased risk of breast cancer.

## Methods

### Study design

**Fig. 1:**
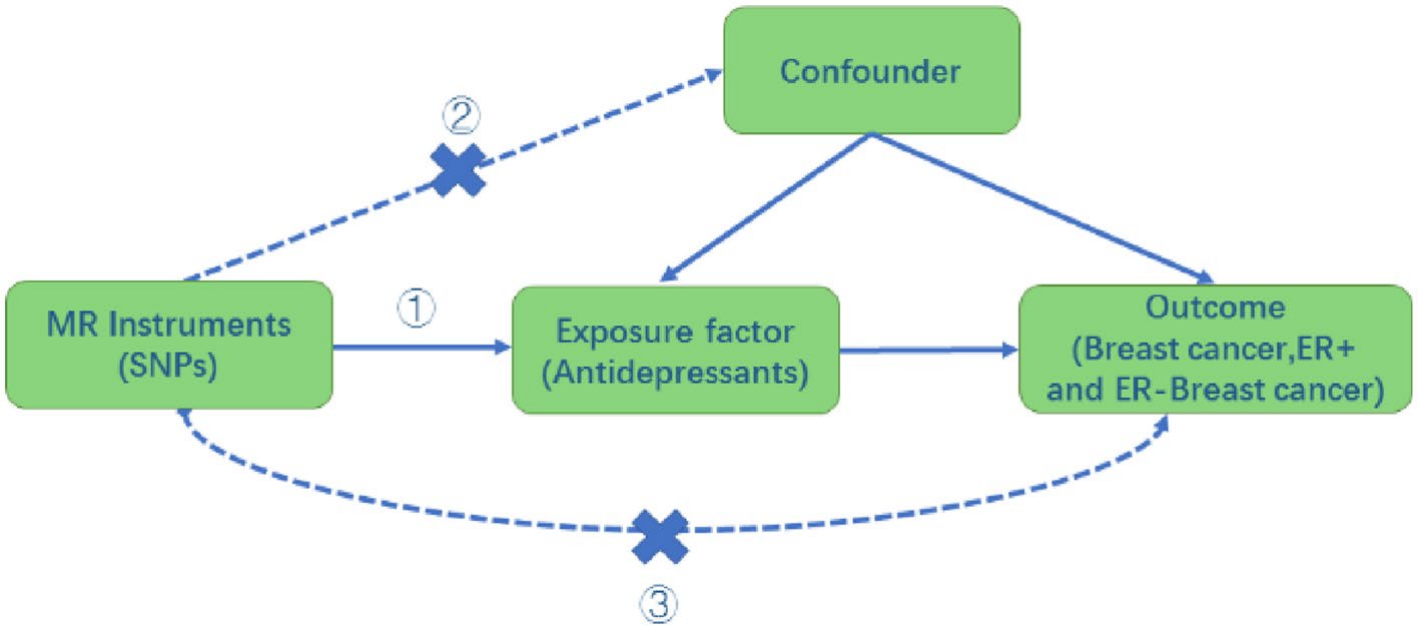
Three conditions that must be met for Mendelian randomization. *Schematic representation of the Mendelian randomization framework. The three core assumptions are as follows: ① SNPs should be closely associated with antidepressants; ②SNP should not be associated with confounders;③ Positive findings will show an impact, all SNPs must be unrelated to breast cancer

**Fig. 2:**
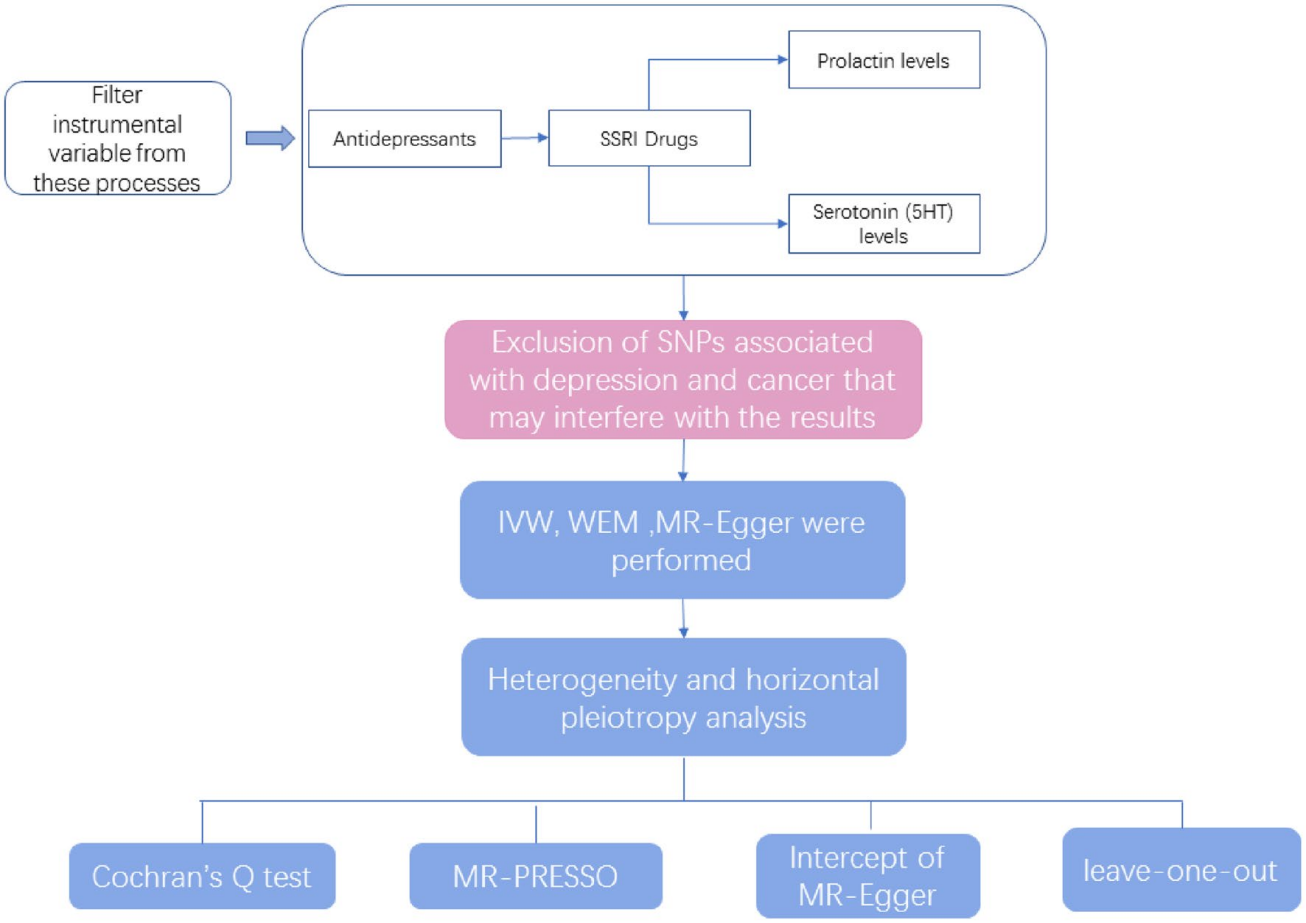
Overview of the study. *IVW, Inverse Variance Weighted; MR-Egger, MR-Egger regression; WME, Weighted Median Estimator; SNP, single-nucleotide polymorphism; MR-PRESSO, Randomization Pleiotropy RESidual Sum and Outlier

This study first conducted an overall analysis of the association between antidepressants and breast cancer risk using the use of antidepressants as an exposure factor. The most controversial SSRIs were then selected as an exposure factor to further investigate the relationship between this class of drugs and breast cancer. We also analyzed the relationship between the risk of breast cancer and the process of increasing prolactin levels by increasing the concentration of 5-HT, which raises prolactin-releasing factor, and the risk of breast cancer by selecting serotonin (5-HT) and prolactin levels as exposures. Finally, the relationship between the use of SSRIs and prolactin levels was explored to see if the use of SSRIs is associated with prolactin levels in the body and thus to investigate whether the SSRI-prolactin-breast cancer hypothesis is valid. All data were derived from single-nucleotide polymorphisms (SNPs) significantly associated with the above exposures as instrumental variables (IVs) and the outcome variable was breast cancer. We performed causal association analysis using a two-sample MR analysis and assessed heterogeneity using the Cochran’s Q test, and finally performed sensitivity analyses to verify the reliability of the causal association results. Additionally, we matched exposure and outcome to determine the direction of the causal effect between exposure and outcome. MR satisfies the following three conditional assumptions: ① there is a strong association between instrumental variables and exposure factors; ② instrumental variables and any confounding of the exposure–outcome association ② instrumental variables are not correlated with any confounding factors of the exposure–outcome association; and ③ instrumental variables do not affect outcome, except possibly through association with exposure. The relationship between the three is shown in Fig. 1. The conceptual diagram of the research design is shown in Fig. 2.

### Sources of information

We selected a GWAS dataset from the FinnGen database that included antidepressant use as an exposure variable. Then to further refine the study, we selected the most problematic SSRIs class of drugs as an exposure factor to examine causality. We selected GWAS data on the use of SSRIs-like medications as exposure from the analysis of the 23andMe database. Finally, we selected two indicators that produced changes after taking SSRIs-like drugs, namely serotonin (5-HT) and prolactin levels, as exposure factors. These data can be found at the following websites and papers: https://www.finngen.fi/fi.; https://gwas.mrcieu.ac.uk/; PMID: 27622933. Outcome data were also obtained from these two sites and included the largest sample of ER-positive breast cancers, the largest sample of ER-negative breast cancers, and the largest sample of breast cancers all from populations of European ancestry in this database.

### Data collation

Our study must satisfy the three conditions of MR, which can be met by collating the data. We satisfied condition 1 by selecting highly correlated SNPs from the exposure data, and the specific parameter was designed to filter out highly correlated SNPs from the exposure GWAS summary data, usually at* P* < 5 × 10^–8^. However, we did not screen enough SNPs in the above dataset, so we downgraded to * P* < 5 × 10^–6^ and * P* < 5 × 10^–7^. Mendelian randomization requires instrumental variables to be highly correlated with exposure, with F > 10 being the strong correlation criterion and F > 10 indicating no weak instrumental variable bias, which is calculated as $F=\frac{N-K-1}{K}\times \frac{{R}^{2}}{1-{R}^{2}},$ where* N* is the sample size of the exposure database,* K* is the number of SNPs, and* R*^2^ is the proportion of variance explained by SNPs in the exposure database. We will calculate F values to ensure that there is no weak instrumental variable bias [17]. To satisfy condition 2, we will extract relevant SNPs from the GWAS summary data of antidepressants, set the linkage disequilibrium coefficient* r*^2^ to 0.001 and the width of the linkage disequilibrium region to 10,000 kb to ensure that each SNP is independent and exclude the effect of gene polymorphism on the results [16]. Secondly, a minimum* r*^2^ > 0.8 was set to replace missing SNPs with highly linked SNPs and to remove SNPs without alternative loci [18]. Finally, the remaining SNPs were searched individually via the Phenoscanner website (http://www.phenoscanner.medschl.cam.ac.uk/) for possible polymorphic effects search, and any SNPs that could have influenced the results in other ways were eliminated. Condition 3 was then tested using methods such as MR-Egger regression and MR-PRESSO to see if the results were influenced by means other than exposure.

### Mendelian randomization analysis

MR analysis is a powerful tool in epidemiological studies. In this research, Inverse Variance Weighted (IVW), MR-Egger regression (MR-Egger), and Weighted Median Estimator (WME) were mainly used for MR analysis. The IVW principle is to weigh each instrumental variables by the reciprocal of the variance of each instrumental variable while ensuring that all instrumental variables are valid [16], and the regression does not take into account the intercept term, the final result is a weighted average of the effect values of all instrumental variables [19]. The WME is defined as the median of the weighted empirical density function of the ratio estimates [20], which provides a consistent estimate of causality if at least half of the valid instruments in the analysis are present.

### Sensitivity analysis and reliability evaluation

Heterogeneity test is mainly to test the difference between individual IVs, if the difference between different IVs is large, then the heterogeneity of these IVs is large, and the large heterogeneity may cause bias to the results of some algorithms, so we usually use Cochran’s Q test to reduce the bias caused by heterogeneity Risk. Cochran’s Q test quantifies the heterogeneity of individual causal effects and is often used to assess whether there is heterogeneity between instrumental variables. If heterogeneity exists between instrumental variables (Cochran’s Q *P* < 0.05), the results are analyzed as IVW for the random effects model, and if not, the results are dominated by IVW for the fixed effects model [21].

The Pleiotropy test primarily tests whether there is horizontal pleiotropy for multiple IVs. In MR studies, instrumental variables are required to affect outcome only through the exposure factor under study, and there is no direct association between instrumental variables and outcome. As genetic variants are multipotent, it is difficult to fully test the exclusivity hypothesis and the intercept term of MR-Egger regression is now commonly used to test for the presence of multipotency. When the linear regression intercept of the MR-Egger model, Egger-intercept, is close to 0, it means that there is no pleiotropy in the instrumental variables and the exclusion hypothesis can be considered valid; otherwise, it means that there is genetic pleiotropy and the exclusion hypothesis is not valid [22, 23]. Mendelian Randomization Pleiotropy RESidual Sum and Outlier (MR-PRESSO) was also used to assess gene pleiotropy and to correct the estimates by removing outliers [24].

We used a “leave-one-out” approach for sensitivity analysis. The combined effect of the remaining SNPs was calculated by phasing out each SNP, observing the effect of each SNP on the results, assessing the effect of each SNP on the results and the stability of the results [16].

All methods were implemented using the TwoSampleMR package in R 4.2.2 software with a test level of *α* = 0.05.

### Ethical considerations

This paper re-analyzes published data and therefore does not require additional ethical approval or ethics approval.

## Results

### Identification of instrument variables

The SNPs screened from the GWAS database above were searched individually on the Phenoscanner and GWAS catalog websites to exclude SNPs associated with breast cancer such as BMI, and then the statistic F was calculated to retain SNPs with an F greater than 10, thus reducing the possibility of bias in the results due to the presence of weak instrumental variables. The remaining SNPs after exclusion will be used for Mendelian randomization analysis.

### Results of Mendelian randomization analysis

The results of this study showed that there was no significant risk association between antidepressant use and breast cancer, and this result was true for both ER-positive and ER-negative breast cancers (All methods *P* > 0.05). Our results also support that SSRI medication is not associated with breast cancer risk (All methods *P* > 0.05) and that this result applies to both ER-positive and ER-negative breast cancers. In addition, the results of the analyses on serotonin (5-HT) and various breast cancers were all methods have p values greater than 0.05. However, it is worth noting that our analysis showed an association between prolactin levels and breast cancer (IVW, OR = 1.058, 95% CI [1.009,1.109], *P* = 0.02) and ER-positive breast cancer (IVW, OR = 1.066, 95 % CI[1.007,1.128], *P* = 0.027), with higher prolactin levels increasing the risk of breast cancer and ER-positive breast cancer. However, in a subsequent analysis of SSRI medication and prolactin, the results showed that taking SSRIs and prolactin levels were not associated (All methods *P* > 0.05). After swapping exposure and outcome, we performed a Mendelian randomization analysis and there was no causal relationship between all exposures and outcome (All methods *P* > 0.05). Bidirectional Mendelian randomization results in individual *P* values greater than 0.05 indicating a stable causal relationship. More details are given in Table 1

**Table 1:** Results of all Mendelian randomization

Exposure	Outcome&ID	Method	Pval	OR^*^	95% CI low	95% CI up
SSRI	ER+ Breast cancer ieu-a-1127	MR-Egger	0.404	0.948	0.844	1.064
		Weighted median	0.315	0.956	0.876	1.044
		IVW^**^	0.431	0.967	0.889	1.052
	ER- Breast cancer ieu-a-1135	MR-Egger	0.825	0.968	0.734	1.276
		Weighted median	0.651	1.058	0.828	1.352
		IVW	0.540	1.061	0.877	1.284
	Breast cancer ieu-a-1126	MR-Egger	0.834	0.988	0.890	1.097
		Weighted median	0.939	1.003	0.935	1.075
		IVW	0.654	0.983	0.911	1.060
Prolactin levels	ER+ Breast cancer ieu-a-1127	MR-Egger	0.421	1.065	0.921	1.231
		Weighted median	0.056	1.070	0.998	1.147
		IVW	0.027	1.066	1.007	1.128
	ER- Breast cancer ieu-a-1128	MR-Egger	0.387	1.122	0.877	1.434
		Weighted median	0.797	1.016	0.900	1.147
		IVW	0.268	1.053	0.961	1.153
	Breast cancer ieu-a-1126	MR-Egger	0.478	1.047	0.927	1.183
		Weighted median	0.118	1.052	0.987	1.120
		IVW	0.020	1.058	1.009	1.109
Antidepressants	ER+ Breast cancer ieu-a-1127	MR-Egger	0.924	1.023	0.661	1.581
		Weighted median	0.100	1.138	0.975	1.328
		IVW	0.167	1.106	0.959	1.275
	ER- Breast cancer ieu-a-1128	MR-Egger	0.363	1.295	0.781	2.146
		Weighted median	0.813	0.975	0.790	1.203
		IVW	0.165	1.123	0.954	1.322
	Breast cancer ieu-a-1126	MR-Egger	0.810	0.947	0.622	1.444
		Weighted median	0.222	1.081	0.954	1.224
		IVW	0.377	1.061	0.930	1.210
Serotonin	ER+ Breast cancer ieu-a-1127	MR-Egger	0.861	1.065	0.539	2.106
		Weighted median	0.465	1.150	0.791	1.672
		IVW	0.143	1.233	0.932	1.630
	ER- Breast cancer ieu-a-1128	MR-Egger	0.836	0.805	0.111	5.822
		Weighted median	0.278	0.609	0.249	1.491
		IVW	0.233	0.640	0.308	1.332
	Breast cancer ieu-a-1126	MR-Egger	0.889	1.044	0.584	1.865
		Weighted median	0.173	1.255	0.905	1.741
		IVW	0.524	1.080	0.853	1.366
SSRI	Prolactin levels ebi-a-GCST90012030	MR-Egger	0.851	0.986	0.871	1.082
		Weighted median	0.842	0.990	0.901	1.091
		IVW	0.809	0.988	0.907	1.116

*OR, odds ratio;**IVW, Inverse Variance Weighted

### Heterogeneity test and sensitivity analysis

We performed Cochran’s Q test for all analyses and the results showed that the p values for the IVW MR-Egger regressions were all greater than 0.05 indicating that there was no heterogeneity in the SNPs. Also the intercept egger_intercept of the MR-Egger’s regression was close to 0 indicating that the results of the causal effects analysis were less likely to be influenced by genetic pleiotropy and the assumption of exclusivity could be considered valid. In the leave-one-out sensitivity analysis, no single SNP strongly influenced the results overall. In addition, the funnel plot provided no evidence of horizontal pleiotropy, and the MR-PRESSO test results, after correction, showed that there was no genetic pleiotropy bias or outliers, so we can assume that there was no horizontal pleiotropy in the SNPs.

## Discussion

Using large-scale GWAS data, this study applied two-sample MR to explore the relationship between antidepressants overall and SSRI drugs, SSRI and prolactin levels, and the causal relationship between 5-HT and prolactin levels and breast cancer risk. The results did not find clear evidence to support a causal effect on breast cancer risk from taking antidepressants or from a corresponding increase in indicators after taking antidepressants. However, we did find that elevated prolactin levels led to an increased risk of breast cancer and ER-positive breast cancer. Interestingly, our study showed that the previously controversial increase in prolactin levels with SSRI drugs was not significant in our analysis, and the SSRI-prolactin-breast cancer hypothesis does not hold up in the light of our findings.

Although our research suggests that antidepressants do not increase the risk of breast cancer, the role of antidepressants in breast cancer risk has been studied for nearly three decades and the relationship between the two has not been fully understood. As early as the 1990s, many researchers found that antidepressants increased the incidence or growth of mammary tumors in rodents through animal studies [25]. There is no consistent evidence on the risk of antidepressants causing breast cancer, and many clinical trials have produced opposite results. In a random-effects meta-analysis that included 18 observational studies, AD use did not increase the risk of breast cancer (RR = 1.02; 95% CI, 0.96–1.08). No difference was observed between SSRI use and breast cancer risk when studies were grouped by drug type (OR 1.02; 95% CI 0.95–1.02) [26]. A nested case–control study that included 19 clinical studies from 6 countries showed no increased risk of breast cancer with antidepressant use (OR = 1.04; 95% CI: 0.97–1.13; *P* > 0.05; *I*^2^ = 74%) and a case–control study (OR = 0.99; 95% CI: 0.93–1.05; *P* > 0.05; *I*^2^ = 33%), a result that holds across a wide range of antidepressants such as TCAs and SSRI and SNRI antidepressants [27], but this study was not stratified by family history, ethnicity, etc. However, there have been contrasting case–control studies showing an increased risk of progesterone receptor-negative and estrogen receptor-positive/PR-negative breast cancer in patients who had taken SSRIs compared to those who had never taken SSRIs (OR = 1.8, 95% CI: 1.1–3.6 and OR = 2.0, 95% CI: 1.1–3.8), but this study was only stratified by family history and breast cancer subtype and did not exclude confounding factors such as BMI that might have an impact, so it is difficult to provide strong evidence [28].

This study has the following advantages. Firstly, because genetic variation is long term and stable and can be measured directly, confounding factors such as social environment and lifestyle are avoided. Secondly, in contrast to randomized controlled trials, Mendelian randomization allows for truly random allocation and is not unethical. Finally, two-sample Mendelian randomization has a relatively larger sample size, allowing for a greater degree of certainty. Our study may provide additional information for clinical decision making, and the results of the study suggest that health professionals may take less account of breast cancer risk when prescribing antidepressant classes to patients.

There are some limitations to this study. Firstly, this study used a population sample of European origin, which lacks data from other ethnic groups, and data from other ethnic groups will need to be analyzed for comparison to make the results more reliable. Secondly, we did not find GWAS data related to the dose of antidepressants, which deprives us of the possibility of further refinement.

In conclusion, this study used two-sample Mendelian randomization to infer a causal relationship between antidepressants and breast cancer and concluded that there was no causal relationship between antidepressants and breast cancer and that there was no relationship between serotonin levels and breast cancer, while taking SSRIs did not raise prolactin levels. However, there is a risk between higher serotonin levels and breast cancer and ER-positive breast cancer. In conclusion our study may provide additional information for clinical decision making and the results of the study suggest that health professionals may take less account of breast cancer risk when prescribing antidepressant-like medications to patients.

## Conclusion

This is the first MR study to explore the causal relationship between antidepressants and breast cancer. The results of our three-method MR analysis concluded that antidepressant medication did not increase the risk of breast cancer, and that although prolactin levels were associated with breast cancer, there was no causal relationship between SSRI use and prolactin levels. Heterogeneity and sensitivity tests validated the robustness of our results.

### Supplementary Information

Below is the link to the electronic supplementary material.

Supplementary file1 (DOCX 16 KB)

## Data Availability

The data that support the findings of this study are openly available in the OpenGWAS database (https://gwas.mrcieu.ac.uk/datasets/; https://www.finngen.fi/fi).
