# Supplementary material for: The relationship between antidepressants and breast cancer: evidence from Mendelian randomization
Source: Cancer Causes Control. 2023 Aug 4;35(1):55–62. doi: 10.1007/s10552-023-01766-z (PMC10764580; doi:10.1007/s10552-023-01766-z)
Supplement: Supplementary file 1 — Supplementary file1 (DOCX 16 KB) [file 10552_2023_1766_MOESM1_ESM.docx]

Table 1 Details of outcome GWAS data

| Outcime | ID | ncase | ncontrol | Sample size | Number of SNPs | Population |
| --- | --- | --- | --- | --- | --- | --- |
| Breast cancer | ieu-a-1126 | 122,977 | 105,974 | 228,951 | 10,680,257 | European |
| ER+ Breast cancer | ieu-a-1127 | 69,501 | 105,974 | 175,475 | 10,680,257 | European |
| ER- Breast cancer | ieu-a-1128 | 21,468 | 105,974 | 127,442 | 10,680,257 | European |

Table 2 Bidirectional Mendelian randomisation results

| Outcome | Exposure | Method | Pval | OR |
| --- | --- | --- | --- | --- |
| 5-HT | ER+ Breast cancer | IVW | 0.45 | 1.11 |
|  | ER- Breast cancer | IVW | 0.06 | 0.98 |
|  | Breast cancer | IVW | 0.77 | 0.96 |
| Depression medications | ER+ Breast cancer | IVW | 0.86 | 1.00 |
|  | ER- Breast cancer | IVW | 0.53 | 0.98 |
|  | Breast cancer | IVW | 0.47 | 1.01 |
| Prolactin levels | ER+ Breast cancer | IVW | 0.16 | 0.97 |
|  | ER- Breast cancer | IVW | 0.97 | 1.00 |
|  | Breast cancer | IVW | 0.17 | 0.97 |
